# The myth of oral hygiene using synthetic mouthwash products

**DOI:** 10.1186/s40064-016-3158-5

**Published:** 2016-09-02

**Authors:** Ghulam Zahara Jahangir, Durre Shahwar Ashraf, Idrees Ahmad Nasir, Madeha Sadiq, Sobia Shahzad, Farah Naz, Muhammad Iqbal, Afifa Saeed

**Affiliations:** 1 Centre for Applied Molecular Biology (CAMB), University of the Punjab, Lahore, Pakistan; 2 Department of Chemistry, GC University, Lahore, Pakistan; 3 National Centre of Excellence in Molecular Biology (CEMB), University of the Punjab, Lahore, 53700 Pakistan; 4 Department of Botany, University of Agriculture, Faisalabad, Pakistan

**Keywords:** Anti-mutagenic, AP sites, DSB, Essential oils, Mouthwash, Comet assay

## Abstract

The synthetic oral cleansing and teeth whitening products like mouthwashes exert adverse effects on teeth, gums and mucous membrane of oral cavity and their extensive use is being criticized. Determining the effect of frequent use of mouthwashes, human cheek cells and their DNA have been studied. Five mouthwash brands were tested and their effects were examined on membrane and DNA of human cheek cells which were found to be very expressive and severe. The DNA, also, received severe damage and breaks developed in its double stranded structure resulting in detachment of small fragments from DNA. The statistical analysis, also, showed significant difference P < 0.005 between the values obtained for DNA double strand breaks for different mouthwashes (and standard mutagen) as compared to untreated control. The study revealed that damage to DNA increases many folds when different mouthwashes are combined. Essential oils of six spice plants (black pepper, clove, black seasam, cinamon, carom seeds and cumin) were evaluated for possessing anti-mutagenic property. These essential oils were found effectively protective against the DNA damaging effect of mouthwashes but could not inhibit it completely. Black pepper, clove, black seasam, cinamon, and cumin were stronger protective as compared to carom seeds.

## Background

With the changing trends and lifestyle the methods of oral hygiene have changed. Now commercial products of oral hygiene, made from synthetic chemicals, are preferred over natural sources and are being commonly used, perhaps because of easy availability and immediate results. But these cleansing and whitening products like mouthwash and toothpaste are being extensively criticized because of their adverse effects on teeth, gums and mucous membrane of oral cavity. One of the most important side effects of mouthwashes is the DNA damage in cheek cells. The cheek cells retain regenerative potential. And, a cell that receives DNA damage but is not detached from the oral mucosa, if not repaired it may cause a mutation. Serious and unrepaired mutations in critical genes can obstruct cell’s ability to perform its normal functioning and thus, can lead to increased danger of swear mouth diseases. There may many reasons of these mutations in cheek cells but frequent application of synthetic products is among the major ones. The mutagenic effect of most products of oral hygiene including some mouthwashes is attributed to the mutagenic property of hydrogen peroxide because it is used as whitening agent in such products. The hydrogen peroxide passes through the dentin and enamel of teeth in return they get change in color. Therefore, in recent years many useful compounds are being identified and isolated from plants which can be added as protective agent (anti mutagen) to the products of oral hygiene to avoid their negative effects on human health. As natural products, the essential oils have been reported for possessing interesting applications (Asbahani et al. 2015).

Present study investigates the (negative) impact of five different mouthwashes on the morphology and DNA of human cheek cells, in vitro; and estimates the protection efficiency of essential oils of spice plants against the mouthwashes. Essential oils of the selected spice plants have extensively reported for possessing antioxidant properties and health benefits; such as cumin (Sowbhagya 2013), clove (Sultana et al. 2014), black sesame (Hosseinzadeh et al. 2007), cinnamon (Moarefian et al. 2013), carom (Singh et al. 2004), and black pepper (Damanhouri and AhmadA 2014). For estimation, the DNA damage posed by mouthwashes was compared with the damage caused by a standard mutagen, the hydrogen peroxide (350 µM). For this study a versatile technique, single cell gel electrophoresis (SCGE), was practicede. SCGE is an efficient tool for studying DNA damage (Li et al. 2012) and is applicable on wide range of cells (Szeto et al. 2005). The abasic sites or apurinic/apyrimidinic (AP) sites in treated cells were counted to identify whether the damage was of oxidative nature. DNA double-strand breaks (DSBs) were also recognized. Former uses an aldehyde reactive probe (ARP) that reacts with an aldehyde group on the open ring at DNA that becomes available for reaction after removal of a nitrogenous base (AP site). Later encounters the phosphorylation of a histone H2A variant, the H2AX, at the sites of break in double stranded DNA.

This study neither criticizes the manufacturers nor the quality of pharmaceutical products of Pakistan at all. The purpose of this study is strictly constructive. It merely finds out the reality hidden behind the modern myth of using mouthwash as an effective choice for oral hygiene and finds that the extensive use of mouthwashes for oral hygiene is not a safe choice. Second focal point of the study is to evaluate the natural extracts (essential oils of spice plants) for anti-mutagenic capability whether it can be suggested after complete study that the mouthwashes and other products formulated for oral hygiene may be modified to add some natural extracts to its formula for protective purposes. The essential oils of the clove, cinnamon, black pepper, black sesame, carom seeds and cumin found possessing DNA protective potential that can be used in mouthwashes to minimize the risk of diseases due to mutagenic effect of such products.

## Methods

### Cheek cell collection

The samples of cheek cells were collected with the consent of randomly selected normal, healthy, nonsmoker male and female students of age between 18 and 25 years belonging to CEMB, University of Punjab, Lahore, Pakistan during their internship program at CAMB in February 2012. The sampling was conducted with the administrative permission and the volunteers were briefed about the purpose of the research before taking their consent. The cells of inner mouth lining were harvested from the mouth waste (water) containing saliva and cheek cells after scratch and swirl activities had performed upon oral mucosa for maximum yield of cells. Then the voluntaries were asked to spit in the clean autoclaved jars and the mouth waste was poured into micro centrifuge tubes (1.5 ml) and cells were pelleted at 5000 rpm at room temperature for 15 min. The cell pellet was washed with autoclaved double distilled water (AddH_2_O) at room temperature at 5000 rpm for 15 min. The washed cell pellet was re-suspended in 100 µl of AddH_2_O and diluted the cell suspension up to required concentration as per requirement of certain experiment.

### Experimental treatments

Mouthwashes (Niflam, Enziclor, Clinica, Prodent and Bannet) were purchased from different medical stores after an informal survey. Hydrogen peroxide 30 % (Biobasic: HC4060) was purchased from Penicon Technologies Pakistan. Experimental treatments were prepared using 100 µl of 10 × 10^5^ concentrated cell suspension in water for each treatment. 10 µl of 350 µM H_2_O_2_ and 10 µl H_2_O were used per 100 µl of cell suspension to prepare the positive control and negative control treatment respectively. Similarly, 10 µl of each of Niflam, Enziclor, Clinica, Prodent and Bannet were used per 100 µl of (10 × 10^5^ concentrated) cell suspension to prepare five separate treatments of mouthwashes. All the treatments were performed in replicates of three. The treatment time was 10 min for every experiment to study the effect of mouthwash except in some experiments of comet assay; in those experiments brief treatment (of 5 min) was given after 24 h for two consecutive days. 1.0 ml of AddH_2_O was added to the treatment mixture immediately after 10 min to obstruct the ongoing activity at once.

#### Treatments with essential oils

During this study, six spice plants have been evaluated for bearing DNA protective potential. The essential information of the studied spice plants are described below in the Table 1. The spices were purchased from a brand store of local market of Lahore, were identified and compared to the record Herbarium by the faculty of the Department of Botany GCU, Lahore. The reference numbers of the record specimens are given in table. All spices were dried under shade and milled to powder form (1 kg) were subjected to steam distillation using Dean Stark apparatus. The crude essential oil of all spices was separated, dried over anhydrous sodium sulphate, filtered and kept in vials at 4 °C and were tested for their efficiency to safeguard the cells against certain mouthwash treatments.

**Table 1:** Essentials details of the plants tested for anti-mutagenic potential

Spice	Local name	Botanical name		Family	Plant part used for essential oil extraction	Identification number
		Genus	Species			
Black pepper	Kali Mirch	*Piper*	*nigrum*	Piperaceae	Fruit	GCU-Herb-Bot-999
Cinnamon	Dar Chini	*Cinnamomum*	*zeylanicum*	Lauraceae	Bark	GCU-Herb-Bot-865
Black sesame	Kalonji	*Nigella*	*sativa*	Ranunculaceae	Seeds	GCU-Herb-Bot-1001
Clove	Long	*Syzygium*	*aromaticum*	Myrtaceae	Buds	GCU-Herb-Bot-985
Carom seeds	Ajwain	*Ptychotis*	*ajowan*	Umbelliferae	Seeds	GCU-Herb-Bot-1003
Cumin	Zeera	*Cuminum*	*cyminum*	Apiaceae	Seeds	GCU-Herb-Bot-989

For the purpose, cheek cells were treated with all mouthwashes and essential oils separately. 70 µl of a mouthwash (taking one at a time) and 30 µl of a certain experimental essential oil (again taking one at a time) making 100 µl in total were mixed well. The clean pelleted cheek cells were re-suspended in above treatments in replicates of three, mixed well, and agitated several times during the treatment period (of 10 min) to constantly keep cells in contact with oil. Autoclaved double distilled water (AddH_2_O) (1.0 ml) was added to the treatment mixture immediately on completion of treatment time so that the activity of both, essential oil and mouthwash could be stopped simultaneously. The tubes containing experimental mixtures were centrifuged immediately after addition of water, at room temperature at 5000 rpm for 15 min, to pellet out the cells.

### Cleaning of cells after treatments

These pelleted cells were washed with 100 µl AddH_2_O at same conditions as above and again re-suspended for further experimentation. This cleaning was enough for the treatments with essential oils only; but an additional step was required to clean the cells after treatment with essential oils as the oil became adhered to the walls of micro tubes during discarding the oil containing supernatant. Therefore, before preceding ahead the walls of micro tubes were cleaned manually with a small spatula having pointed tip and wrapped with autoclaved thin paper of blotting capability to maintain the quality of the experiment. The concentration of the cell suspension was maintained using Neubauer Improved MARIENFELD Heamocyto meter which was attached to OLYMPUS CKX41 microscope.

### Study of the cheek cell morphology

The smears of treated cells were prepared, after above described cleaning process, on sterilized microscopic slides by taking a drop of cell suspension. Control slide was prepared from cells without any treatment of mouthwash. The drop of cell suspension was spread with the help of a disposable cell scraper. The smear was glued over the slide by passing over the flame of spirit lamp several times. Each fixed smear was stained by a drop of Safranin (Merck: 109217 Gram’s safranine solution, supplied by Merck Pakistan) for 1 min. After rinsing the excessive color the slides were elaborately studied and captured at 20× and 40× magnification (Olympus BX61 DP Controller).

### Study of the DNA of cheek cell

To identify and estimate the impact of mouthwash and/or essential oils on the DNA of cells experimented under different treatments, following methods were adopted.

#### Comet assay or single cell gel electrophoresis technique (SCGE)

It was used as a tool to identify whether the individual cells had received any negative impact of mouthwash treatments and counter effect of essential oils’ treatments. SCGE protocol was followed using some reagents of OxiSelect™ Comet Assay Kit (STA-350) and it was purchased from FY Diagnostics and Surgical Pakistan.

#### Oxidative DNA damage

For quantification of damage, the DNA was extracted from the treated, cleaned cells using Norgen Saliva DNA Isolation kit (50, Catalog Number 45400) which was purchased from Penicon Technologies Pakistan. Isolated pure DNA was quantified with Nanodrop Spectrophotometer ND-1000 and saved at 4 °C until used. Its concentration was adjusted according to the need of the protocol just before experiment. Then DNA damage was quantified using OxiSelect™ Oxidative DNA Damage (AP Sites) Quantitation Kit (Catalog Number STA-324) in accordance with the step by step protocol detailed in it. The kit was purchased from FY Diagnostics and Surgical Pakistan.

#### Gel electrophoresis of DNA

The DNA extracted from different treatments (as mentioned in “Oxidative DNA damage” section) was run along with 1 kb DNA ladder (fermentas) at 4 V/cm on 1.0 % high melt agarose gel (prepared in TAE buffer) to find the DNA damaging effect of treatments. In another experiment a bulk of DNA was extracted (as mentioned in “Oxidative DNA damage” section) from negative control (untreated) cells and following treatments were applied on it.

- All five mouthwashes alone for 10 min (1 µl treatment per 10 µl DNA in TE buffer).
- All six essential oils with every mouthwash (1 µl mouthwash, 1 µl essential oil per 10 µl DNA in TE buffer).

#### Double strand breaks in DNA

The protocol of OxiSelect™ (Catalog Number STA-321) DNA double strand break (DSB) Staining Kit was modified according to the requirement of cheek cells. The kit was purchased from FY Diagnostics and Surgical Pakistan.

##### Protocol

The cheek cells were harvested, prepared, treated, and washed as described in above sections of methodology. Then the cell pellet was re-suspended into AddH_2_O up to 5 × 10^5^, poured 100 µl per well of the 96-well plate. Put at room temperature for 15 min. Added 100 µl of diluted DNA DSB Inducer (provided in kit) to each well and incubated for 1 h at 37 °C. Carefully removed the liquid from cells then cells were fixed by gently adding 100 µl 3.7 % Formaldehyde/PBS per well of the plate and incubated the plate for 30 min at room temperature (to allow the cells to settle down and get fixed to the plate). Fixed cells were washed once (very gently) with 200 µl of 1× PBS. The wells were aspirate and 100 µl of ice-cold 90 % Methanol was added per well and incubated at 4 °C for 15 min. Fixed cells were washed again (once) with 200 µl of 1× PBS. After aspirating the wells 200 µl of Blocking Buffer was added to each well and incubated at room temperature for 30 min on orbital shaker. Again aspirated the wells and 100 µl of 1× anti-phospho-histone antibody solution was added to each well and incubated on orbital shaker at room temperature for 1 h. The wells were washed five times with 200 µl wash buffer (PBST). 100 µl/well of 1× secondary antibody (and 100 ml of 0.002 mg/ml ethidium bromide (Et Br) in distilled water was used for staining) was added after aspirating the previous solution and again incubated on orbital shaker at room temperature for 1 h. The cells were washed again three times with 200 µl/well of Wash Buffer (PBST). After aspirating 200 µl of 1× PBS was added to each well. The cells were analyzed under TRITC filter of fluorescence microscope for presence of DSBs.

#### Statistical analysis

The data was evaluated statistically by analysis of variance (one way ANOVA) and its POST HOC test using SPSS software version 20. The means followed by different letters within each column are significantly different at P < 0.005.

## Results

### Effect of mouthwash on the morphology and DNA of cheek cells

#### Effect on membrane of cheek cells

**Fig. 1:**
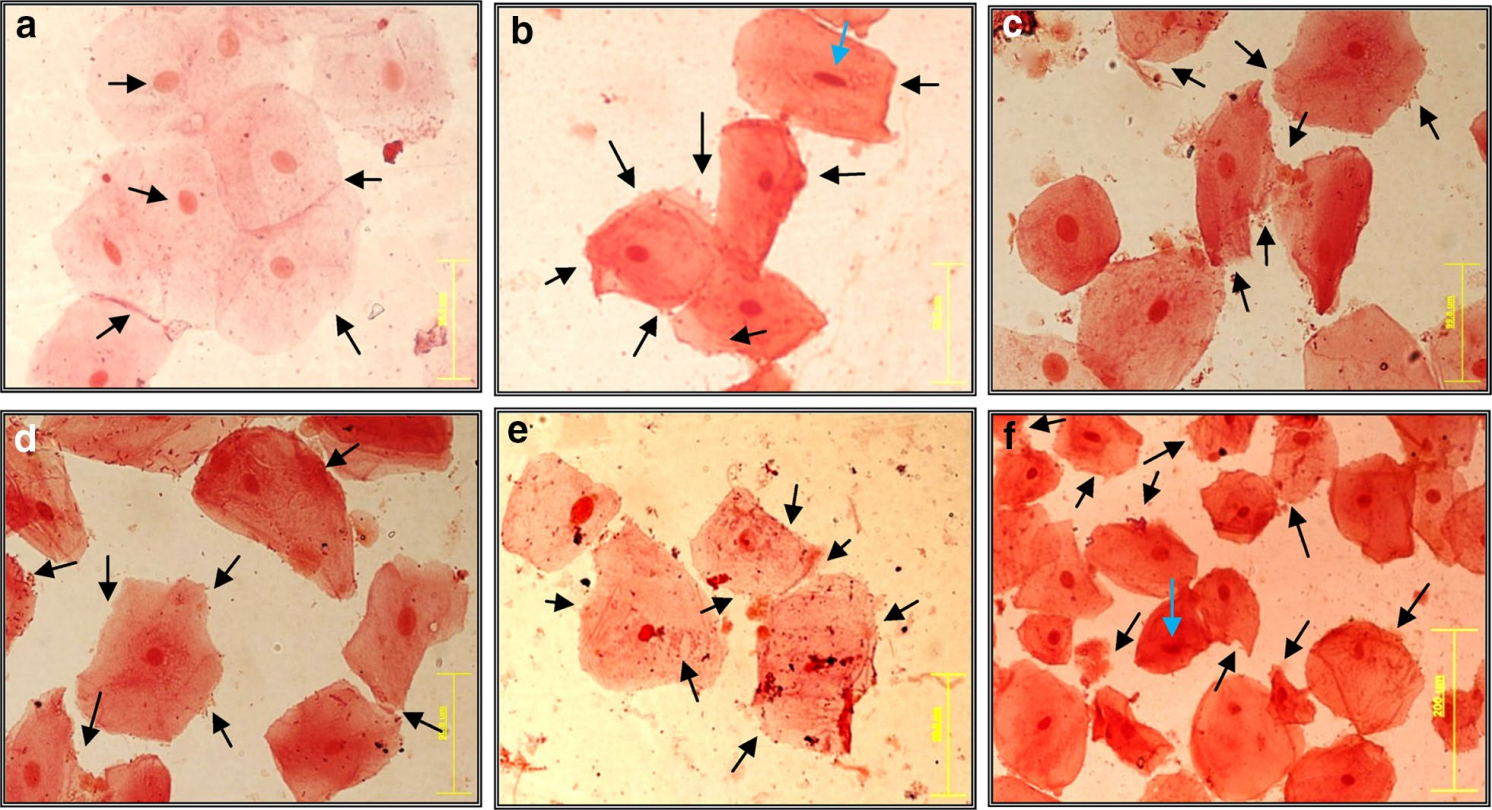
Effect of mouthwashes on the morphology of human cheek cells. **a** Negative control, **b** Niflam, **c** Prodent, **d** Clinica, **e** Bannet and **f** Enziclor (×40 magnification of Olympus BX61 DP Controller)

The non-treated cells in smear (of negative control slide) attained fresh pink color and were observed as larger, apparently healthy and turgid with clearly visible undisturbed nucleus and granular cytoplasm at 40× of magnification (Fig. 1a). Whereas, the treated cells were found damaged with cuts in their outer membranes and all the treated cells absorbed too much stain on mounting and almost similar observations were observed with all five treatments (each with six replicates). The cells treated with Niflam, Prodent, Clinica, and Bannet received deep cuts on their plasma membranes (Fig. 1b–e respectively). Enziclor treated cells showed less deep cuts in membranes (Fig. 1f) but their nuclei became squeezed, elongated and diameter of cells appeared to be reduced comparative to the normal cheek cells (Fig. 1a, f respectively). Thus, the mouthwashes not only enhanced the porosity of the cell membranes but also damaged them by generating deep cracks that caused loss of cellular fluid and allowed the enhanced passage of materials through the cell membranes. However the extent of severity varied for different mouthwash treatments.

#### DNA damaging effect of mouthwashes

**Fig. 2:**
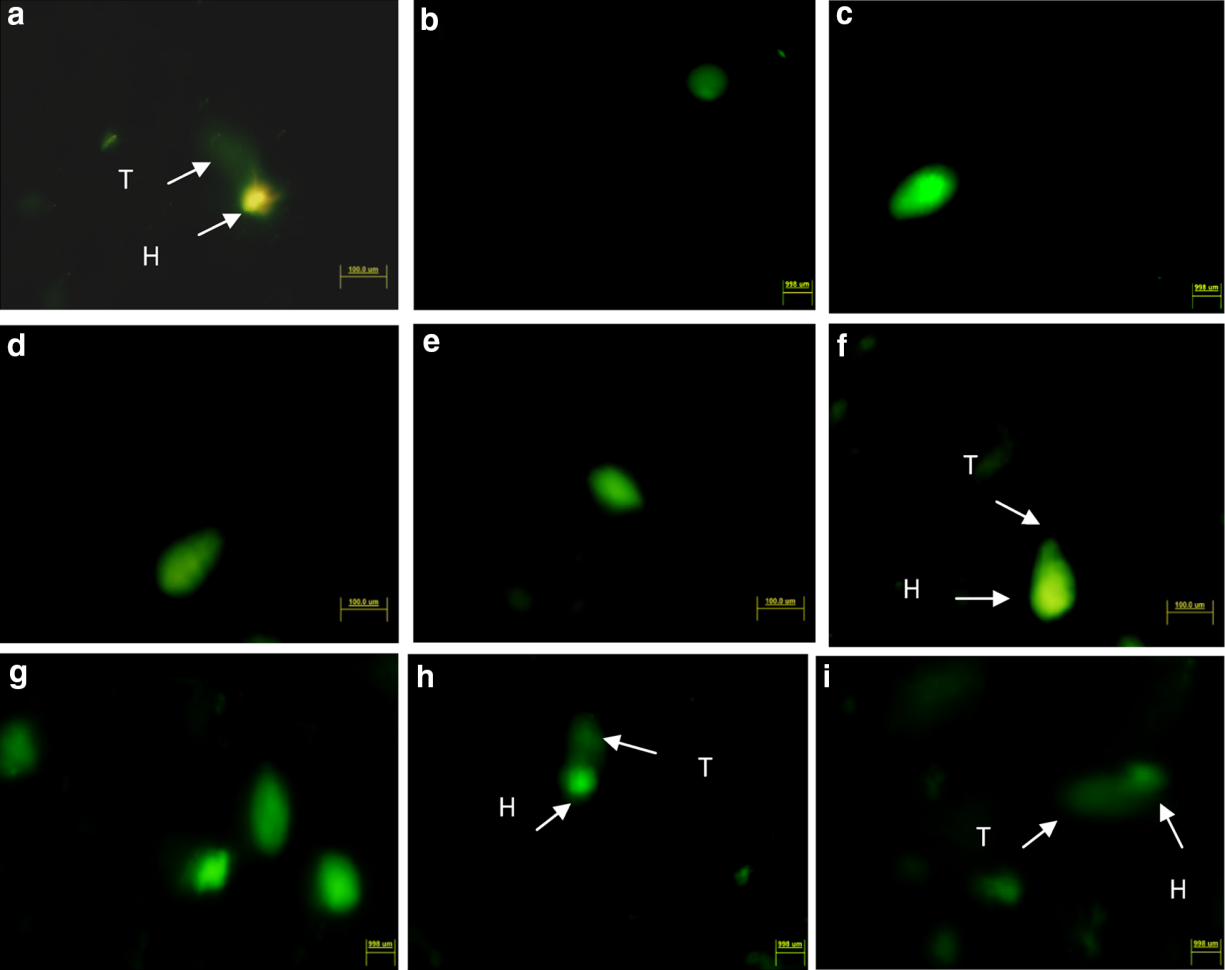
Comet assay: effect of mouthwashes on human cheek cells. **a** +ve control, **b** −ve control, **c** Niflam, **d** Prodent, **e** Clinica, **f** Bannet, **g** Enziclor, **h** double treatment with Enziclor, **i** double treatment with Bannet (Vista Green, FITC filter, ×20 magnification of Olympus BX61 DP Controller)

Morphological study of cheek cells gave sufficient evidence about the negative impact of mouthwashes on them. Therefore, comet assay technique was performed to identify their effect on DNA and comparatively more severe effect was observed. The results are summarized in Fig. 2. Nuclei of positive control (350 µM H_2_O_2_) replicates produced typical comets with long tails (Fig. 2a) whereas circular nuclei were observed in all replicates of negative control (neither treated with mutagen nor mouthwash Fig. 2b). All mouthwashes showed comparative effect on DNA of cheek cells exhibiting prominently damaged nuclei in all replicate slides of Niflam, Prodent, Clinica, Bannet, and Enziclor (Fig. 2c–g respectively). The slides with brief but twice treatment of mouthwashes (Fig. 2h, i) received more DNA damage than single treatment of 10 min and produced long tailed comets that can be compared with those formed in positive control (Fig. 2a). The results supported the concept that the mouthwashes are strong enough to pass through the membranes of cell and nucleus; approach the DNA of cheek cells and cause significant damage. Although the H_2_O_2_ was not mentioned as an active ingredient of any of the mouthwash but all showed almost similar impact when treated on cheek cells. Thus the mouthwashes must have some quantity of H_2_O_2_ and if not it has some other chemical that is strong enough to affect the integrity of cheek cells and its DNA as the H_2_O_2_ does.

### Quantitaion of the DNA damage

So far the potential of mouthwash to cause DNA damage has evaluated. The extent of DNA damage was also determined using following methods.

#### Quantitation by counting AP sites

**Fig. 3:**
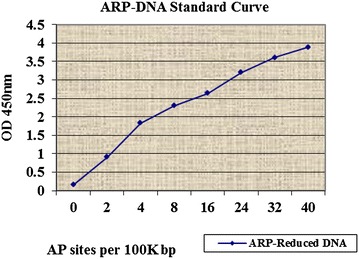
Absorbance of ARP-reduced standard DNA at 450 nm

Absorbance of different concentrations of ARP-Reduced Standard DNA and different treatments at 450 nm of micro spectrophotometer (Molecular Devices Spectra Max Plus 384) are recorded in Table 2. Theoretically OD_450_ is positively related to the number of AP sites hence the oxidative DNA damage. A standard curve was obtained from recorded optical densities (OD) of ARP-reduced standard DNA at 450 nm which served as a standard control (Fig. 3). No relation was observed in the recorded OD_450_ for different treatments (Table 3) whereas these treatments caused a significant DNA damage (Fig. 2). For all treatments the number of AP sites was not more than one as almost all absorbance fell in the area of standard curve between 0 and 2 (Table 3). From these results no conclusion could be derived however two possibilities were very apparent. First, the methodology or the proceedings were faulty and second that AP sites were not formed (in DNA on treating with mouthwashes); hence the mouthwash had not caused the oxidative damage. First possibility lacks firm ground and can be easily eliminated as the methodology was authentic and OD_450_ of standards was in exact accordance to the expected one (Fig. 3).

**Table 2:** Preparation of ARP-DNA standards

Tubes	ARP-DNA standard (µl)	Reduced DNA standard (µl)	TE buffer (µl)	Total volume (µl)	DNA concentration (µg/ml)	AP sites per 100,000 bp
1	20	0	100	120	1	40
2	16	4	100	120	1	32
3	12	8	100	120	1	24
4	8	12	100	120	1	16
5	4	16	100	120	1	8
6	2	18	100	120	1	4
7	1	19	100	120	1	2
8	0	20	100	120	1	0

**Table 3:** Absorbance against different treatments for determination of AP sites

Well number	OD of ARP + reduced DNA standard from Table 2	Theoretically AP sites per 100,000 bp	Different treatments and their absorbance			
1	3.888	40	−ve control 0.800	Bannet 0.44	Niflam 0.156	Niflam 0.331
2	3.60	32	Enziclor 0.718	Bannet 0.293	Niflam 0.525	Clinica 0.672
3	3.20	24	Enziclor 0.69	Bannet 0.74	Niflam 0.34	Clinica 0.35
4	2.633	16	Enziclor 0.74	Prodent 0.231	Niflam 0.65	Clinica 0.591
5	2.633	8	Enziclor 0.66	Prodent 0.65	–	Clinica 0.291
6	1.83	4	Enziclor 0.73	Prodent 0.39	–	Clinica 0.314
7	0.912	2	Bannet 0.384	Prodent 0.47	–	–
8	0.152	0	Bannet 0.41	Prodent 0.56	–	–

#### Quantitation by gel electrophoresis

**Fig. 4:**
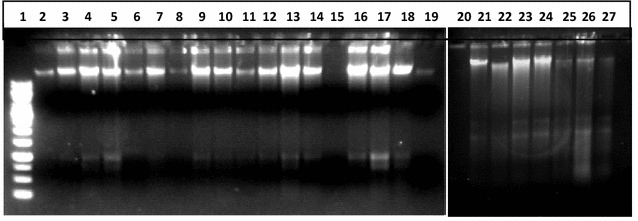
DNA damaging effect of mouthwash on human cheek cells. *1* 1000 bp DNA ladder, *2* negative control: DNA from untreated cells, *3*–*7* DNA from cells treated with Enziclor, *8*–*12* DNA from cells treated with Bannet, *13*–17 DNA from cells treated with Prodent, *18*–*22* DNA from cells treated with Niflam, *23*–*27* DNA from cells treated with clinica

The same DNA to verify either the DNA was broken into fragments because of mouthwash treatments. DNA from all treatments was run on agarose gel. When studied under ultraviolet light, it was observed to produce DNA fragments who were found on gel distant away from genomic DNA; and many of them were smaller than 2000 bp. DNA of three samples (cheek cells) out of five treated with Bannet, did not show fragmentation (well numbers 3, 6,7). Similarly the DNA of two samples out of five treated with Niflam (well numbers 19 and 22) did not produce fragments and their DNA bands were similar to the DNA of control sample in well number 2 (Fig. 4). This may be attributed to the high immunity of an individual and even if it is because of less perfection of the handling the results are reliable because only to avoid such exceptions five replicates were processed simultaneously. In the DNA of cells treated with Enziclor, Bannet, Prodent, and Niflam fragments of 1000 bp (or slightly larger) got detached from whole genomic DNA. While the DNA of cells treated with Clinica produced two smaller DNA fragments of 2000 and 1500 bp respectively (Fig. 4).

**Fig. 5:**
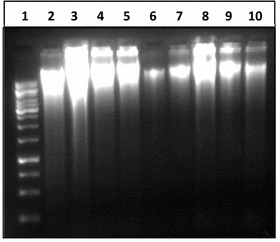
Effect of mouthwash on human white blood cells. *1* 1000 bp ladder, *2* −ve control (without any treatment), *3* treatment with Bannet, *4* clove essential oil and Bannet, *5* treatment with Enziclor, *6* clove essential oil and Enziclor, *7* treatment with Prodent, *8* clove essential oil and Prodent, *9* treatment with Clinica, *10* clove essential oil and Clinica

The experiment was also performed on the human white blood cells taking clove essential oil as protective agent against all five mouthwashes separately. But it found quite difficult to induce mutagenesis in human blood cells and the DNA remained absolutely intact even after 20 min of treatment (Fig. 5). This quality might be attributed to high level of immunity of white blood cells that either nullified the effect of mouthwashes or hindered them to reach the nuclei of the cells.

**Fig. 6:**
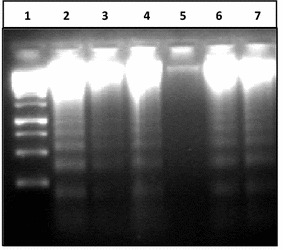
Mutagenic effect of mouthwashes on human DNA. *1* 1000 bp DNA ladder, *2* DNA treated with Enziclor, *3* DNA treated with Bannet, *4* DNA treated with Prodent, *5* negative control: untreated DNA, *6* DNA treated with Niflam, *7* DNA treated with Clinica

On treating purified DNA with mouthwashes, isolated from untreated cells, it broke into pieces of different sizes and an array appeared on the gel (Fig. 6). The smallest fragment was of <250 bp as it moved away even from the last band of 1 kb DNA ladder (250 bp). All other prominent fragments were smaller than 1000 bp and the approximate quantity of broken DNA was very small as compared to main largest molecule of DNA. In every treatment six smaller DNA fragments detached (and moved farther) from the DNA molecule. The mutagenic effect of mouthwashes on naked DNA molecule was more severe (Fig. 6) than on DNA when it was protected inside Nucleus (Figs. 4, 5).

#### Quantitation by recognizing double strand breaks

**Fig. 7:**
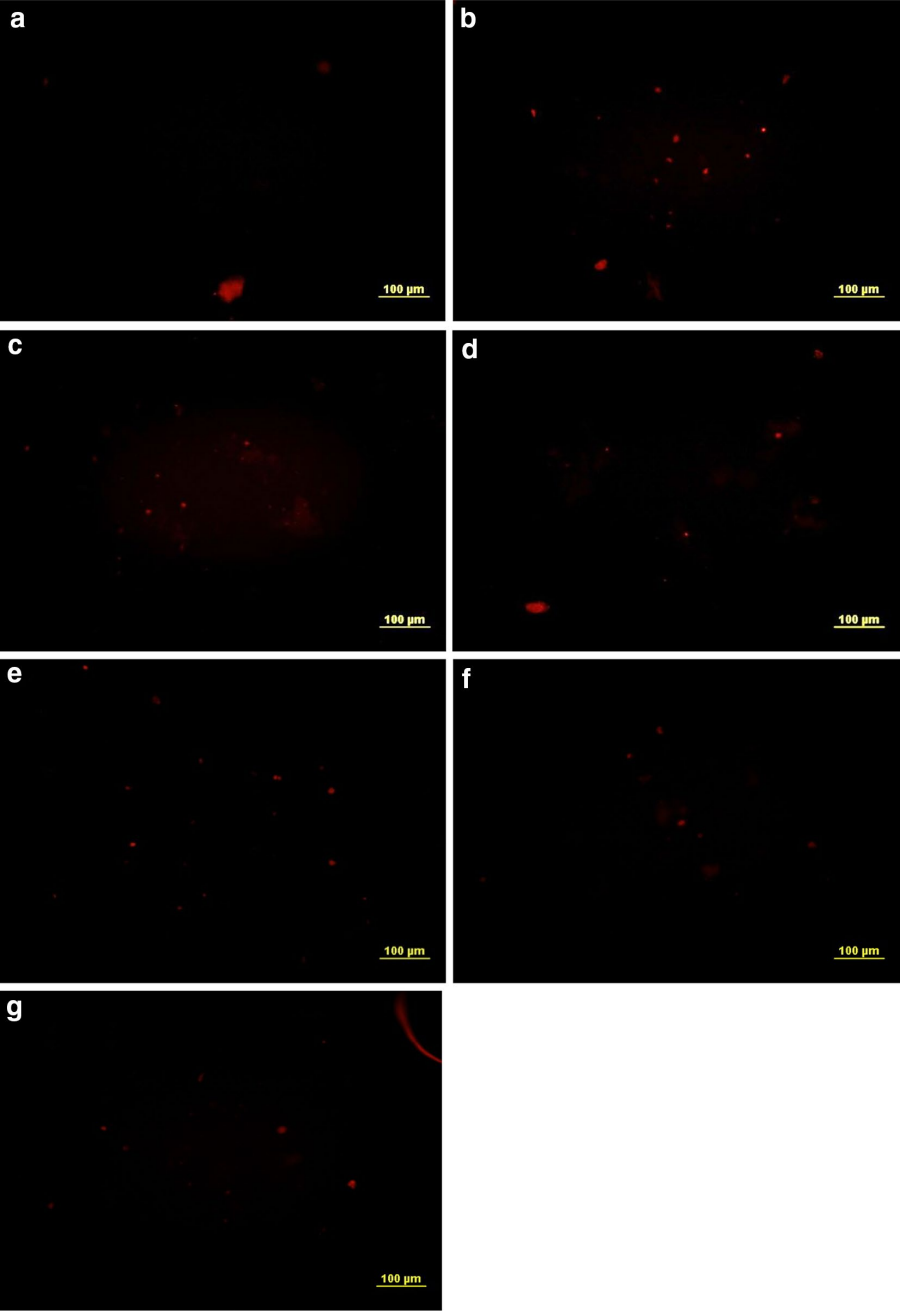
Mouthwash treatments develop DNA double strand breaks in human cheek cell. **a** Negative control, **b** positive control (350 μl H_2_O_2_), **c** Bannet, **d** Enziclor, **e** Niflam, **f** Prodent, **g** Clinica (**c**–**g** double treatments) (Et Br, TRITC filter, of Olympus IX51, 96-well ELISA plate)

Previous studies showed that mouthwashes do not produces AP (apurinic and/or apyrimidinic sites) sites in DNA which is the most common kind of oxidative DNA damage. But when DNA from treated cheek cells was electrophoresed very clear fragments were recorded. For further verification treated cheek cells were tested for possessing breaks in double strands of DNA and found decisive results that are summarized in the Fig. 7. Untreated cheek cells (negative control) did not produced fluorescence under TRITC filter which shows that phosphorylation of a histone H2A variant (H2AX) has not happened; hence the breaks in double strand of DNA has not produced (Fig. 7a). A lot of fluorescent sites were observed in the slides prepared from cheek cells which were treated with standard mutagen, the positive control (Fig. 7b), and from cells with mouthwash treatments (Fig. 7c–g). From statistical analysis, also, significant difference P < 0.005 was observed between the values obtained for different mouthwashes and standard mutagen as compared to untreated control (Table 4). These observations lead to the conclusion that the mouthwashes must have H_2_O_2_, although it was not mentioned in its active ingredients, that causes DNA damage just as H_2_O_2_ and if not it has some other chemical that is strong enough to affect the integrity of DNA as H_2_O_2_ does.

**Table 4:** Statistical analysis of treatments with mouthwashes for DNA double strand breaks

Serial no.	Treatments	Days		
		1st day	2nd day	3rd day
1	Control	3.0 ± 0.5^b,g^	4.0 ± 0.00^b,f^	4.0 ± 0.00^b,c,d,f,g^
2	Prodent	16.0 ± 0.577^a,c–f^	10.0 ± 0.577^a,f^	13.0 ± 0.577^a,c,d–f^
3	Bannet	5.3 ± 0.666^b,g^	7.0 ± 0.00^f^	10.0 ± 0.577^a,b,e,f,g^
4	Clinica	7.0 ± 0.577^b,g^	8.6 ± 0.577^f^	10.6 ± 0.577^a,b,e–g^
5	Enziclor	7.0 ± 0.00^b,g^	6.0 ± 0.577^f^	5.0 ± 0.00^b–d,f,g^
6	Niflam	6.0 ± 0.577^b,g^	20.0 ± 0.00^a–e,g^	7.0 ± 0.00^a–e,g^
7	Standard mutagen	16.0 ± 1.73^a,c–f^	11.0 ± 0.00^a,f^	15.0 ± 0.00^a–f^

### Protection efficiency of essential oils

**Fig. 8:**
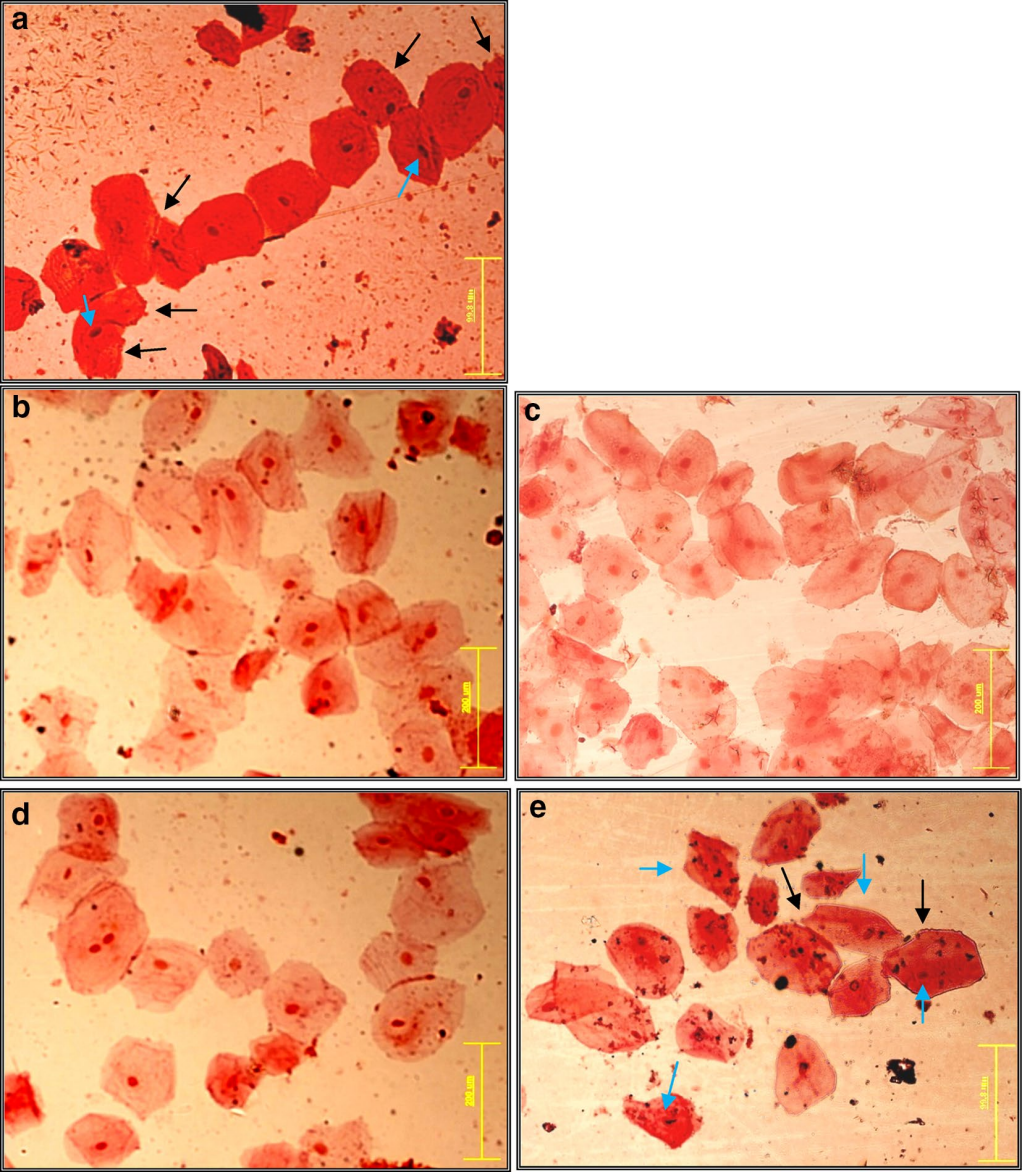
Efficiency of essential oils to protect cheek cells against mouthwashes. **a** Control smear, **b** black pepper +Prodent, **c** cinnamon + Bannet, **d** black seasam + Niflam, **e** carom seeds + Enziclor (×20 magnification of Olympus BX61 DP Controller)

Remarkable defense against mouthwashes was observed for all of the tested samples of essential oils of spice plants. The cells of control treatment, which were not treated with essential oil but with mouthwashes only, absorbed intensive stain on mounting and received many cuts on their membranes (Fig. 8a). Smears from essential oils and mouthwash combinations attained less color upon mounting (Fig. 8b–d). All the treatments of essential oils showed very good protection to cells, against the corrosive effect of mouthwashes except essential oil of carom seeds (Fig. 8e) as the cells absorbed extensive stain and some cells also got squeezed as compared to normal cells (Fig. 1a). The squeezed cells are pointed with blue arrows in Fig. 8a, e.

**Fig. 9:**
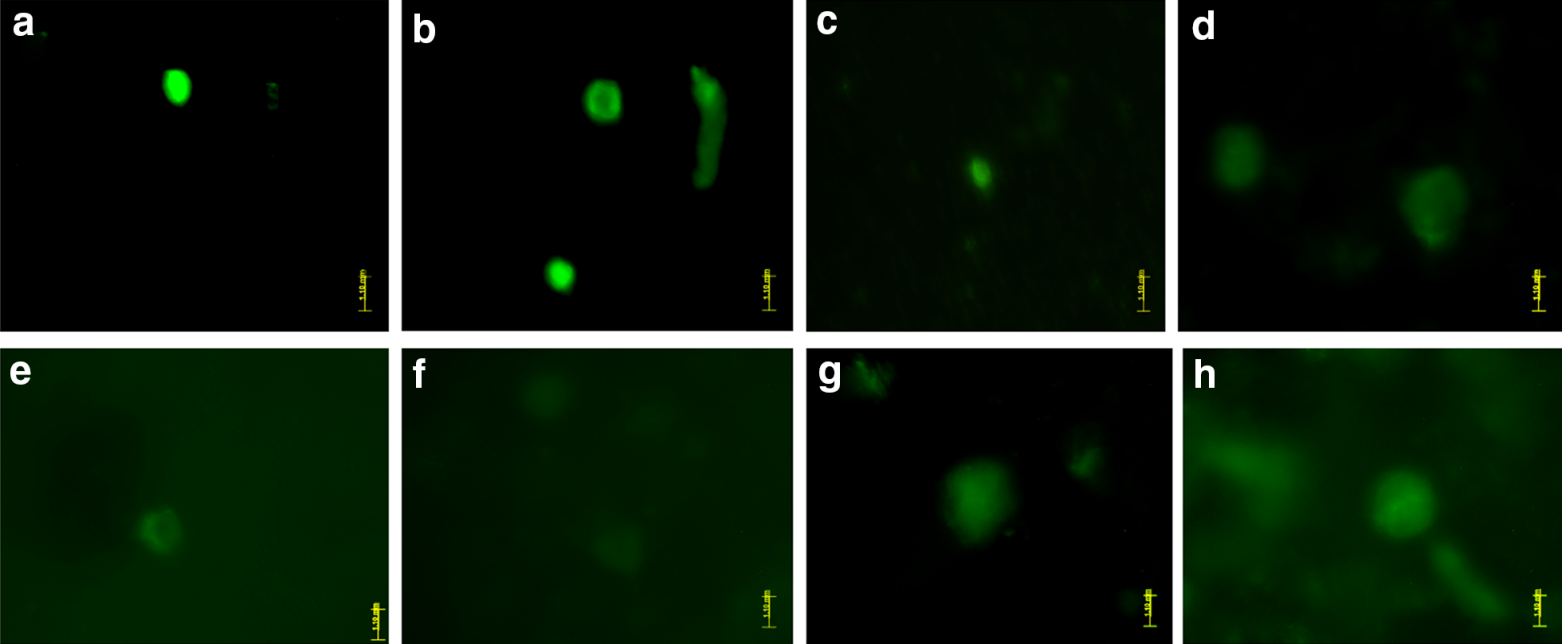
Comet assay; capability of essential oils to counter DNA damaging effect of mouthwash. **a** Clove against Bannet, **b** black seasam against Enziclor, c black pepper against Bannet, **d** Cinnamon against Clinica, **e** carom seeds against Prodent, **f** cumin against Niflam, **g** clove against Enziclor, **h** black seasam against Clinica (**a**–**c** ×20 and **d**–**h** ×40 magnification of Olympus BX61 DP Controller), *vista green*, FITC filter

In other experiments to test the defense potential of essential oils against DNA damaging effect of mouthwashes, essential oils effectively protected the nuclei from mouthwashes and no significant DNA damage was observed in any replicate slide prepared from the cells that were treated with mouthwash and essential oils in combination (Fig. 9a–h). However, the nuclear boundaries of cells from these treatments were not found intact as was observed in negative control cells (Fig. 2b) nor any prominent comet was found as was in the case of single and double mouthwash treatments (Fig. 2c–i). Thus, it can be said that essential oils of spice plants effectively protect DNA from damage but cannot inhibit it completely.

**Fig. 10:**
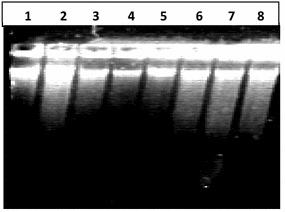
Anti mutagenic effect of essential oils on human DNA. *1* Negative control (untreated DNA), *2* positive control (DNA treated essential oil only), *3* DNA treated with Enziclor and clove, *4* DNA treated with Bannet and clove, *5* DNA treated with Prodent and clove, *6* DNA treated with Niflam and clove, *7* DNA treated with Clinica and clove, *8* DNA treated with Clinica and cinnamon

Protective efficiency of essential oils (of clove and cinamon) for DNA molecule was also tested and Fig. 10 summarizes the results. It was found that plant extracts protected the DNA from the mutagenic effect of mouthwash and the DNA bands remained intact and were similar to the band of DNA of control (Fig. 10). The wells of gel are glowing because of the presence of essential oils of treatment as the DNA was loaded directly after treatment to avoid any effect of purifying solutions on DNA structure.

## Discussion

### Mutagenic effect of mouthwashes

In the presented study hydrogen peroxide was used in different concentrations as a standard mutagen to induce DNA damage in positive control treatments. It is used as whitening agent in most products meant for oral hygiene including some mouthwashes. The DNA damage posed by standard mutagen was compared with the damage caused by mouthwashes. The methodology of the presented study coincides with work of many other researchers like Miranda-Vilela et al. (2010), Szeto et al. (2005), and Donnelly et al. (1999) who used hydrogen peroxide in various concentrations to induce DNA damage.

Cheek cells were found as an effective model to study the impact of mouthwashes as the sampling was easy and cheap, and also because the buccal mucosa is at direct stake of chemical harm posed by products of oral hygiene. The complete and uniform lysing of the plasma membrane was found difficult in the case of cheek cells. The time of treatment in all experiments was not more than 10 min because a person rinses his mouth with a mouthwash approximately for about 1.5 min a day. On average it makes approximately 10 min a week. This is the lower limit as the “Directions to Use” on the cover of many mouthwashes advise minimum rinse of 2–3 min. This makes exposure of cells in oral cavity up to minimum of 14–20 min a week. Szeto et al. (2005) selected buccal cells as model for comet assay on nutritional and bio monitoring studies in vitro (Szeto et al. 2005). Kuyama and Yamamoto (1997) studied the influence of mouthwashes on the human oral mucosae and found it negative if used daily (Kuyama and Yamamoto 1997). They also selected 10 min time span to study the impact and used exfoliative cytological and cytomorphometric analyses to assess the impact of mouthwashes. As they studied the impact in vivo, they took samples before and after use of mouthwash. They also found reduce in the nuclear and cytoplasmic areas of cells and inflammations in cells even after 1 h of mouthwash use. Their findings are in close accordance with those presented in this work (Fig. 1b, f).

The comet assay technique was found effective to study the DNA damaging effect of mouthwashes and to evaluate the protective efficiency of spice plants extracts. The methodology and finding (Figs. 2, 9) coincides with those of Szeto et al. (2005) who found comet assay as the best option among all other methods to study the DNA damage (Szeto et al. 2005). Zaika et al. (2011) used comet assay to study the DNA damage while they were investigating the role of p73 protein in DNA damage (Zaika et al. 2011). Li et al. (2012) used OxiSelect™ Comet Assay Kit (STA-350) to study the effect of protein–protein interaction on DNA damage to study the behavior of poly-SUMO chain inhibitor and the role of gold nanoparticles for the creation of multivalent poly-SUMO (Li et al. 2012). Donnelly et al. (1999) also practiced modified alkaline SCGE (comet assay) to determine DNA integrity using hydrogen peroxide to induce DNA damage while they were finding the effect of ascorbate and α-tocopherol, both singly and in combination, against induced DNA damage and reactive oxygen species (Donnelly et al. 1999).

The methodology of the presented study (Fig. 3; Tables 2, 3) is in accordance to the methodology selected by Zaika et al. (2011) who quantified oxidative DNA damage by counting the AP sites through the Oxiselect™ Kit and found that its deficiency increases the damage (Zaika et al. 2011).

Findings of the presented study reveal that frequent use of mouthwashes is not safe for the cells of oral mucosa. A lot of the reported research reveals similar opinion and puts strong criticism over the extensive use of synthetic mouthwashes. For instance Wynder et al. (1983) examined the role of mouthwash and other factors in relation to oral cavity cancer by means of a retrospective study. According to their research daily use of mouthwash showed an excess risk in females but no excess risk in males and no dose response was seen in females with increased duration of use. In nonsmoking, nondrinking women as well, daily mouthwash use was associated with excess risk (Wynder et al. 1983). Winn et al. (1991) also observed increased risks of cancer associated with the regular use of mouthwash in a study on 866 patients with cancer of the oral cavity and pharynx and 1249 controls of similar age and sex in the general population in four areas of the United States.

### Anti mutagenic effect of essential oils

Essential oils of spice plants were found protective against mutagenic effect of mouthwashes. The methodology and findings are in harmony with those of Jayakumar and Kanthimathi (2012) who tested cumin, black pepper and clove extracts for anti-mutagenic activity through SCGE technique by using hydrogen peroxide as chemical mutagen to induce mutagenesis. Cumin, black pepper and clove extracts, among six other spices, were tested for antimutagenic capability. Clove and pepper were found significantly effective against DNA damage at low concentrations but other spices showed anti mutagenic effect only at high concentration of their water extract (Jayakumar and Kanthimathi 2012). Their findings are in harmony of the methodology and results reported in this document.

Jayaprakasha et al. (2007) found anti mutagenic activity of water extract of cinnamon fruit (Jayaprakasha et al. 2007). Hamssa et al. (2003) found that bell pepper and black peppers possess anti-mutagenic activity. They further stated that both are capable of inhibiting carcinogen activation, improving the detoxification of carcinogens by scavenging the reactive agents that damage DNA. They revealed that black pepper show antimutagenic effect due to inhibition of certain mutagen formation and their direct interaction with electrophilic species (Hamssa et al. 2003). Parveen and Shadab (2012) studied antioxidant activity of spices and found that *Nigella sativa* effectively controls the number of chromosome aberrations induced by chlorambucil (CLB). They further revealed that *N. sativa*’s anti carcinogenic activity inhibits DNA damage but it cannot completely protect cells from damage (Parveen and Shadab 2012). These findings provide evidence to the results reported in this document that *N. sativa* helps to prevent DNA damage.

## Conclusion

This study concludes that the extensive use of mouthwashes for oral hygiene is not safe. The chemical composition of mouthwash is such that it causes damage in the cell structure and DNA of cheek cells. Mouthwashes cause damage to DNA by breaking its double stranded structure. This work suggests that the natural plant extracts are effective source of anti-mutagenic materials. Essential oils of the clove, cinnamon, black pepper, black seasam, carom seeds and cumin possess antioxidant potential and can be used in mouthwashes as protective chemical to avoid or at least to minimize the risk of diseases due to mutagenic effect of such products. Furthermore, SCGE is a good and reliable technique that can be used to study the DNA damaging effect of mouthwashes on human cheek cells because this technique is very versatile and easily modifiable to fulfill the need of certain experiment. However more studies at gene level are required to identify the loci on human genome that receive nicks from mouthwashes. It may lead to find a correlation between the different mouth diseases and use of mouthwash.
